# Combined Treatment with L-Carnitine and Nicotinamide Riboside Improves Hepatic Metabolism and Attenuates Obesity and Liver Steatosis

**DOI:** 10.3390/ijms20184359

**Published:** 2019-09-05

**Authors:** Kanita Salic, Eveline Gart, Florine Seidel, Lars Verschuren, Martien Caspers, Wim van Duyvenvoorde, Kari E. Wong, Jaap Keijer, Ivana Bobeldijk-Pastorova, Peter Y. Wielinga, Robert Kleemann

**Affiliations:** 1 Department of Metabolic Health Research, The Netherlands Organization for Applied Scientific Research (TNO), 2333 CK Leiden, The Netherlands (K.S.) (E.G.) (F.S.) (W.v.D.) (I.B.-P.) (P.Y.W.); 2 Human and Animal Physiology, Wageningen University, 6708 WD Wageningen, The Netherlands; 3 Department of Microbiology and Systems Biology, The Netherlands Organization for Applied Scientific Research (TNO), 3704 HE Zeist, The Netherlands (L.V.) (M.C.); 4 Metabolon Inc., Morrisville, NC 27560, USA; 5 Department of Vascular Surgery, Leiden University Medical Center, 2333 ZA Leiden, The Netherlands

**Keywords:** obesity, non-alcoholic fatty liver disease, β-oxidation, mitochondria, metabolomics, acylcarnitines, transcriptomics, lipid peroxidation

## Abstract

Obesity characterized by adiposity and ectopic fat accumulation is associated with the development of non-alcoholic fatty liver disease (NAFLD). Treatments that stimulate lipid utilization may prevent the development of obesity and comorbidities. This study evaluated the potential anti-obesogenic hepatoprotective effects of combined treatment with L-carnitine and nicotinamide riboside, i.e., components that can enhance fatty acid transfer across the inner mitochondrial membrane and increase nicotinamide adenine nucleotide (NAD+) levels, which are necessary for β-oxidation and the TCA cycle, respectively. Ldlr −/−.Leiden mice were treated with high-fat diet (HFD) supplemented with L-carnitine (LC; 0.4% *w*/*w*), nicotinamide riboside (NR; 0.3% *w*/*w*) or both (COMBI) for 21 weeks. L-carnitine plasma levels were reduced by HFD and normalized by LC. NR supplementation raised its plasma metabolite levels demonstrating effective delivery. Although food intake and ambulatory activity were comparable in all groups, COMBI treatment significantly attenuated HFD-induced body weight gain, fat mass gain (−17%) and hepatic steatosis (−22%). Also, NR and COMBI reduced hepatic 4-hydroxynonenal adducts. Upstream-regulator gene analysis demonstrated that COMBI reversed detrimental effects of HFD on liver metabolism pathways and associated regulators, e.g., ACOX, SCAP, SREBF, PPARGC1B, and INSR. Combination treatment with LC and NR exerts protective effects on metabolic pathways and constitutes a new approach to attenuate HFD-induced obesity and NAFLD.

## 1. Introduction

Worldwide, the prevalence of overweight and obesity in both adults and children has increased dramatically over the last decades and has become a major health concern [1]. Adiposity and ectopic fat accumulation are characteristics of obesity that can cause comorbidities. In recent years non-alcoholic fatty liver diseases (NAFLD) has emerged as the most common chronic liver disease associated with obesity [2]. Current concepts for treatment of obesity focus on lifestyle changes and include dietary advice combined with exercise. Despite lifestyle and dietary advice, obesity rates have not declined in the last three decades [1]. Also, current treatments are insufficient to accomplish sustained weight loss for the majority of obese patients [3]. Hence, there is a need for new strategies that stimulate energy metabolism in other ways [4].

L-carnitine (LC) plays an important role in oxidative metabolism, since it is required for the transfer of long-chain fatty acids (FAs) from the cytosol into the mitochondrial matrix, where β-oxidation occurs. These FAs need to be activated as acyl-CoAs and transformed into acylcarnitines to be transported across the mitochondrial membrane. Acylcarnitines < C22 in length can enter the mitochondrial matrix [5], in exchange for free carnitine, where they are reconverted to acyl-CoAs and can be used for β-oxidation [6]. Similarly, carnitine is also required for the transport of end products of peroxisomal β-oxidation, medium and short-chain acyl-CoAs, out of the peroxisomes to allow further processing in the mitochondria [7]. A decline in carnitine levels has been associated with insulin resistance and diet-induced obesity and was suggested to be a consequence of long-term lipid overload, dysfunction of energy metabolism and incomplete fat oxidation [8]. Conversely, LC supplementation in obese rats was shown to restore carnitine levels and improve metabolic function [8].

Nicotinamide ribose (NR) is a dietary precursor molecule of nicotinamide adenine nucleotide (NAD+) and has been shown to enhance oxidative metabolism and protect mice against high-fat diet (HFD) induced obesity [9]. NAD+ provides reducing equivalents that fuel oxidative phosphorylation, crucial for oxidative metabolism and metabolic homeostasis. It has been shown that NAD+ levels decline in obesity and closely related metabolic disorders, such as diabetes and NAFLD [10,11], suggesting that treatments which enhance NAD+ content may moderate the development of these disorders. NAD+ has also been shown to play a role in limiting oxidative stress damage [10]. Oxidative stress has been implicated during obesity and in human NAFLD development [12]. Several studies have reported increased levels of 4-hydroxynonenal (4-HNE), a marker of oxidative stress-induced lipid peroxidation [13] in NAFLD patients [14,15,16].

The properties of LC and NR that improve oxidative metabolism and enhance FA utilization prompted us to study their effects in a chronic model of high-fat diet-induced obesity, insulin resistance and NAFLD. More specifically, we evaluated the long-term effects of combined LC and NR treatment as a novel strategy for reducing obesity and NAFLD and used respective mono-treatments as a reference. To do so, Ldlr−/−.Leiden mice received HFD with and without LC, NR and their combination (COMBI) for 21 weeks. At this time point the Ldlr−/−.Leiden mice developed obesity and hepatic steatosis, the main endpoints of this study. Moreover, this diet-induced mouse model recapitulates specific molecular metabolomic and transcriptomic signatures of NAFLD patients [17,18,19]. Body composition, plasma metabolic parameters and acylcarnitines, hepatic histology, lipid peroxidation and genome-wide liver transcriptomics were analyzed. These analyses were used to evaluate combined and individual effects of LC and NR on obesity, NAFLD development and hepatic oxidative stress-related damage.

## 2. Results

### 2.1. Experimental Diets Induce Carnitine and Nicotinamide Plasma Levels 

**Figure 1:**
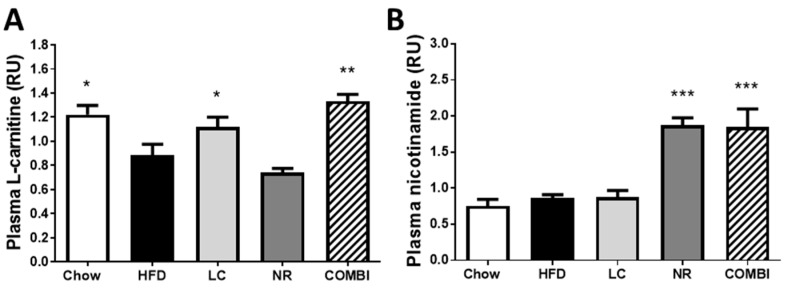
Dietary L-carnitine (LC), nicotinamide riboside (NR) or both (COMBI) supplementation to a high-fat diet (HFD) increased the plasma levels of L-carnitine and nicotinamide after 21 weeks of treatment, indicating effective delivery via the diet. LC-MS was used to measure plasma levels of (**A**) L-carnitine and (**B**) nicotinamide. Data are presented in relative units (RU) as mean ± SEM, * *p* < 0.05 or ** *p* < 0.01 or *** *p* < 0.001 compared to the HFD control group.

Plasma L-carnitine levels were higher in chow than in the high-fat diet (HFD) treated mice (1.2 ± 0.1 RU vs 0.9 ± 0.1 RU; *p* < 0.05) (Figure 1A). Plasma L-carnitine levels in the NR group (0.7 ± 0.0 RU) were comparable to the HFD control group. L-carnitine treatment in the LC and the COMBI groups restored carnitine levels to 1.1 ± 0.1 RU (*p* < 0.05) and 1.3 ± 0.1 RU (*p* < 0.001), respectively, i.e. comparable to the chow healthy reference group.

Plasma nicotinamide concentrations, a metabolite of NR were similar in the chow, HFD and LC groups (0.8 ± 0.1 RU; 0.9 ± 0.0 RU and 0.9 ± 0.1 RU, respectively) (Figure 1B). Nicotinamide riboside treatment in the NR and COMBI groups significantly elevated plasma nicotinamide levels to 1.9 ± 0.1 RU and 1.9 ± 0.3 RU, respectively (both *p* < 0.001).

Overall, these data show that dietary supplementation with LC and NR in an HFD resulted in a normalization or modest increase of the respective circulating metabolites, indicating effective delivery into the circulation. 

### 2.2. Combined Treatment with L-Carnitine and Nicotinamide Riboside Attenuates HFD-Induced Obesity Independent of Food Intake or Activity

To evaluate the effect of LC and NR and their combination on the development of obesity, food intake (FI), activity and body weight (BW) were monitored during the study, and fat mass was determined by EchoMRI at set time points. 

**Figure 2:**
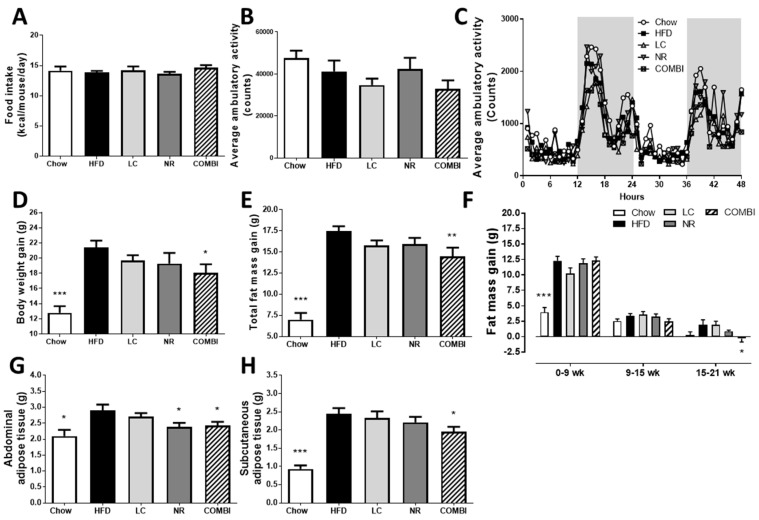
Effect of LC, NR and COMBI treatment on obesity after 21 weeks of treatment. (**A**) Food intake, (**B**) average ambulatory activity represented in counts, i.e., amount of infrared beam interruptions caused by movement, (**C**) average ambulatory activity during 48 hours, (**D**) body weight gain, (**E**) total fat mass gain, (**F**) fat mass gain over time, (**G**) abdominal tissue mass and (**H**) subcutaneous adipose tissue mass. Data are presented as mean ± SEM, * *p* < 0.05 or ** *p* < 0.01 or *** *p* < 0.001 compared to the HFD control group.

Average FI during the study (Figure 2A) was similar in all groups. Ambulatory activity was measured during 48 h in metabolic cages, in which the counts are a measure for beam interruptions caused by movement (Figure 2B,C). These results showed no significant differences in activity between the groups. 

Even though FI and activity were similar, the average BW gain in the HFD control group was 21.4 ± 0.9 g after 21 weeks, which indicated the development of obesity. By contrast, the average BW gain in the chow group was only 12.8 ± 0.9 g (*p* < 0.001) (Figure 2D). Mono-treatment with LC or NR affected BW only slightly, and the BW gain was comparable in these groups (19.7 ± 0.7 g and 19.3 ± 1.4 g, respectively). Notably, BW gain was significantly reduced in the COMBI group (18.1 ± 1.1 g; *p* < 0.05) compared to HFD, indicating that combination therapy attenuated obesity development.

Consistent with this observation, the total gain in fat mass in the HFD group was 17.5 ± 0.6 g whereas mice in the chow group gained significantly less fat (7.0 ± 0.8 g; *p* < 0.001) (Figure 2E). LC and NR alone resulted in 10% reduction in fat gain (15.8 ± 0.6 g; *p* = 0.13 in the LC group and 15.9 ± 0.7 g; *p* = 0.17 in the NR group). COMBI treatment resulted in 17% reduced fat gain (14.5 ± 1.0 g; *p* < 0.01). A more refined longitudinal analysis with EchoMRI revealed that the COMBI treatment was most effective in attenuating fat mass in the period between 15 weeks and 21 weeks (Figure 2F) when obesity was already established. Consistent with the EchoMRI data, the weight of the abdominal white adipose tissue (WAT) depots were significantly reduced by COMBI treatment (Figure 2G), and similar fat mass-lowering effects were observed for the subcutaneous WAT depot (Figure 2H). 

These data demonstrate that COMBI treatment significantly attenuated HFD-induced obesity and adiposity independent of food intake or activity, an effect that could not be achieved with the individual components. 

### 2.3. COMBI Treatment Attenuates Metabolic Risk Factors and Liver Integrity Marker ALT

Compared to the HFD group, fasting plasma cholesterol, high-density lipoprotein (HDL)-cholesterol and non-HDL cholesterol, triglycerides, insulin, blood glucose and alanine aminotransferase (ALT) levels were not significantly lowered with the treatments, although the COMBI treatment had the most reducing effects on these metabolic risk factors (Table 1). We subsequently performed an extensive plasma metabolomics analysis to profile plasma acylcarnitines, which inform on the effects related to fatty acid processing and β-oxidation. 

We observed a significant increase in long-chain acylcarnitines in plasma of HFD-treated mice compared to the chow group (Table 2), indicating an elevation of these lipid species in HFD-induced obesity. When compared to HFD, LC and COMBI treatments resulted in a shift towards more medium-chain acylcarnitines, while NR consistently lowered plasma acylcarnitines concentrations (all values < 0). 

Collectively, these data show that metabolic risk factors and liver integrity marker ALT were partially yet not significantly reversed by LC and NR and the most pronounced lowering effects were observed in the COMBI treatment group.

### 2.4. COMBI Treatment Reduces HFD-Induced Liver Steatosis

**Figure 3:**
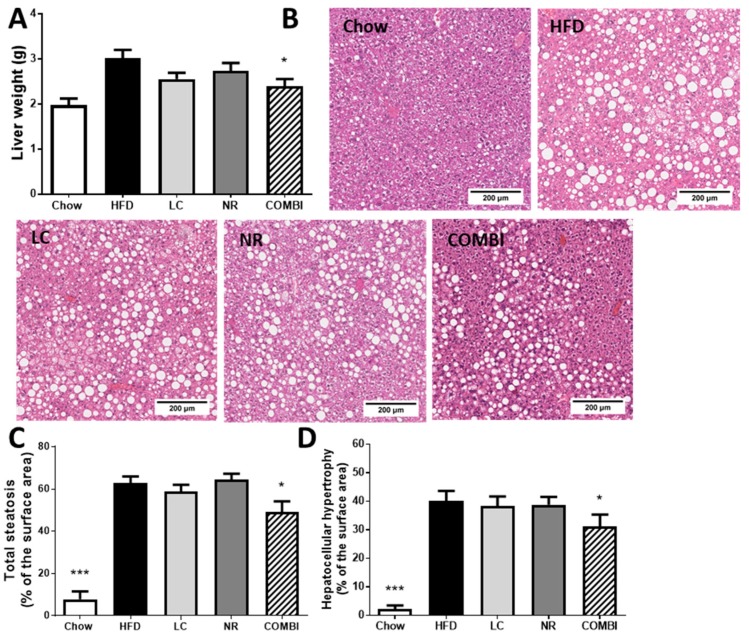
Liver analysis after 21 weeks of LC, NR and COMBI treatment. (**A**) Liver weight, (**B**) representative images of hematoxylin-eosin stained liver sections, (**C**) total steatosis, (**D**) hepatocellular hypertrophy. Data are presented as mean ± SEM, * *p* < 0.05 or ** *p* < 0.01 or *** *p* < 0.001 compared to the HFD control group.

Next, we examined whether the adiposity-attenuating effects of the COMBI treatment had consequences for ectopic fat deposition in the liver. Liver weight was 3.0 ± 0.2 g in the HFD group and 2.0 ± 0.1 in the chow group (*p* < 0.001) (Figure 3A). COMBI treatment significantly reduced liver weight (2.4 ± 0.2 g; *p* < 0.05), which suggested an effect on hepatic steatosis while the mono-treatments had a less pronounced effect (LC 2.6 ± 0.1 g; *p* =0.07, and NR 2.7 ± 0.2 g; *p* = 0.29). To investigate this in more detail, cross-sections of livers were analyzed for histopathological features of NAFLD (Figure 3B) and scored using a human-based grading system as described in the Materials and Methods section. 

Steatosis in the HFD treated mice contained on average 62.8 ± 3.0% of the cross-sectional area, whereas the chow group hardly displayed steatosis (7.7 ± 3.7%; *p* < 0.001) (Figure 3C). The mono-treatments did not affect total steatosis (58.7 ± 3.3%; *p* = 0.18 in LC and 64.3 ± 2.8%; *p* = 0.32 in the NR groups), while COMBI treatment significantly reduced steatosis by 22% (49.1 ± 5.0%; *p* < 0.05). 

Consistent with the steatosis-inducing effect of HFD, abnormally enlarged hypertrophic hepatocytes covered 40.0 ± 3.6% of the cross-sectional area in the HFD group while such cells were hardly observed in mice on the chow diet (2.1 ± 1.3%; *p* < 0.001) (Figure 3D). Neither LC nor NR affected HFD-induced hypertrophy (LC 38.3 ± 3.4%, and NR 38.9 ± 2.7%), but the COMBI treatment showed a significant reduction in hepatocellular hypertrophy (31.1 ± 4.2% of the liver area; *p* < 0.05). 

Collectively, these data show that the adiposity-attenuating effects of the COMBI treatment are associated with a reduction in NAFLD development. 

### 2.5. NR and COMBI Attenuate Hepatic Lipid Peroxidation 

**Figure 4:**
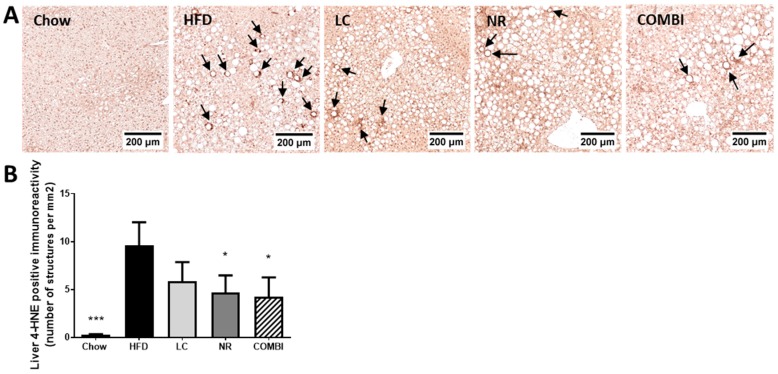
The 4-hydroxynonenal (4-HNE) marker for oxidative stress-related lipid peroxidation was analyzed in the liver using immunohistochemistry after 21 weeks of LC, NR and COMBI treatment. Stained liver sections were used for (**A**) representative images of 4-HNE, and quantification of (**B**) 4-HNE-positive immunoreactivity indicated here by arrows per mm^2^. Data are presented as mean ± SEM, * *p* < 0.05 compared to the HFD control group.

Next, we investigated whether the observed hepatoprotective effects may be associated with an attenuation of oxidative stress induced lipid peroxidation. To this end we used 4-HNE as a marker and quantified the amount of 4-HNE positive structures by immunohistochemistry (Figure 4A,B).

The amount of positive 4-HNE structures in the HFD treated mice was high (9.6 ± 2.4 per mm^2^), whereas the chow group scarcely showed any 4-HNE structures (0.3 ± 0.1; *p* < 0.001). LC only demonstrated a trend in lowering 4-HNE immunoreactivity (5.9 ± 2.0; *p* = 0.09), while both the NR and COMBI treatment revealed a significant decrease in 4-HNE immunoreactivity (4.7 ± 1.8 and 4.3 ± 2.0; both *p* < 0.05).

In conclusion, NR and COMBI treatment significantly attenuated the amount of 4-HNE adducts, suggesting that these treatments could reduce oxidative stress-related lipid peroxidation.

### 2.6. COMBI Counteracts Effects of HFD on Metabolic Pathways in the Liver

A genome-wide gene expression profiling analysis was performed to gain insight into the molecular processes underlying the observed hepatoprotective effects of the COMBI treatment. HFD treatment resulted in 3914 differentially expressed genes (DEGs) compared to chow. The significant changes were used as input for an upstream regulator analysis, as described in the Materials and Methods section. HFD treatment strongly and significantly activated upstream regulators of chemokine and cytokine signaling (e.g., CCL5 and CXCL2, IL1b, IL4, IFNγ); transcriptional inflammatory control (e.g., AP1, JNK, NFκB); TRL signaling (e.g., TRL2, TRL4); metabolic control and hepatic insulin signaling (e.g., ACOX1, INSIG1, INSR, PPARGC1B, FOXO1, TP53, SIRT2) and oxidative stress response (e.g., NOS2, SOD) (Supplementary Material, Table S1). 

LC resulted in 175 DEGs, NR in 208 DEGs and the COMBI treatment had the greatest effect on gene expression with 332 DEGs compared to HFD (Supplementary Material, Figure S1). A subsequent upstream regulator analysis showed that the majority of the above-mentioned regulators were not significantly affected by the mono-treatments (indicated by n/a in Table 3). By contrast, COMBI reversed numerous HFD-induced upstream regulators linked to metabolic control and insulin signaling (Table 3). For instance, HFD inhibited the enzyme ACOX1, involved in the first step of the peroxisomal fatty acid β-oxidation pathway, which was reversed by COMBI treatment. Similar counter-regulatory effects were found for regulators involved in lipid metabolism (SCAP, SREBF1, SREBF2), glucose uptake (SIRT2) and insulin signaling (INSR), which were inactivated by HFD and activated in the COMBI group. Together, this analysis demonstrates that COMBI treatment counteracted the detrimental effects of HFD in the liver and mainly affected master regulators relevant to metabolic control, thereby providing a rationale for its hepatoprotective effects. 

## 3. Discussion

The objective of the present study was to evaluate the anti-obesogenic and hepatoprotective effects of a novel treatment using a combination of LC and NR. We found that dietary supplementation with LC and NR was efficient because it elevated L-carnitine and nicotinamide plasma levels. Furthermore, the COMBI treatment attenuated HFD-induced body weight gain and total fat mass. The observed reduction in obesity development in the COMBI group was independent of food intake or locomotor activity, and was associated with an attenuation of metabolic risk factors and liver integrity marker ALT. COMBI treatment also reduced total steatosis, lipid peroxidation associated damage and affected several upstream regulators involved in liver metabolism. Hardly any of these anti-obesogenic or hepatoprotective effects were observed with the mono-treatments. Studies investigating combinations of LC and NR have not been reported so far, therefore, we used literature describing the effects of the individual components to discuss our findings. 

Measures of adiposity were significantly reduced in the COMBI group. Interestingly, longitudinal Echo-MRI measurements showed that COMBI treatment reduced fat mass development specifically after week 15 (between 15–21 weeks), i.e., when obesity was already established. Since prolonged periods of nutrient excess result in a gradual accumulation of lipids, which may impair mitochondrial metabolism [12] at a certain stage, it is possible that carnitine and NAD+ become essential at the later timepoints when mitochondrial dysfunction and associated oxidative stress is more likely to occur. Indeed, there was pronounced 4HNE-positive immunoreactivity in HFD livers at the end of the study, and this immunoreactivity was lower in the COMBI group indicating reduced oxidative stress-associated lipid peroxidation [13]. We found that mono-treatment with LC did not result in a significant reduction in body weight and fat mass, and similar findings were reported by others studying body composition in rodents [20,21,22] or BMI in healthy and obese humans [23]. Also, short time studies using NR up to 15 weeks in rodents [20,24] and up to 12 weeks humans [25,26,27,28] did not report effects on body weight or body composition. In contrast, Canto et al. and Gariani et al. showed that 12 and 18 weeks of NR 400 mg/kg supplementation in C57Bl/6J mice reduced body weight due to a reduction in fat mass [9,29], respectively. Consistent with these reports, we observed a significant reduction in abdominal fat mass by NR in the present study. However, we did not found effects on total body fat mass, which could potentially be a consequence of a lower NR concentration used in our study.

We did not observe significant effects of the mono-treatments and the COMBI treatment on fasting total cholesterol, triglycerides, glucose and insulin, still the COMBI group showed consistently lower levels in all metabolic risk factors and plasma ALT. In particular the trend towards lower insulin and glucose suggests an effect on insulin signaling. Indeed, hepatic upstream regulator gene analysis predicted that HFD treatment significantly reduced glucose uptake (SIRT2) [30] and insulin receptor activity (INSR), and also revealed the counter-regulatory effects of the COMBI treatment on both processes, suggesting an improvement in the sensitivity of hepatic insulin. 

Both LC and NR effects on plasma metabolic risk factors may be dose dependent. LC with 2000 mg daily for 24 weeks resulted in lower glucose and plasma lipid levels in NASH patients [31] as well as improved insulin sensitivity [32], whereas 19 months treatment with a very low dose of 100 mg/kg LC daily in mice did not reveal any significant effects on plasma lipid levels [33]. Similarly, studies in mice reported lowering effects of 400 mg/kg NR on ALT [27], fasting glucose and an improvement in insulin sensitivity [9,29]. By contrast, a very recent human study employing 2000 mg NR daily supplementation, which is approximately 2–3 times lower in concentration compared to the animal studies, could not reproduce the positive effects on insulin sensitivity and whole-body glucose metabolism in obese insulin resistant men [26]. 

Plasma acylcarnitines are often increased in obese patients with type 2 diabetes [34] and in high-fat diet induced animal models [8,35,36]. Acylcarnitines are typically formed in the cytosol to shuttle FAs across the mitochondrial membrane into the matrix, thereby providing substrate for β-oxidation. The observation that acylcarnitines are elevated in plasma of subjects with metabolic disease suggest that in the case of chronic oversupply with FAs, the capacity to further process these FAs has been reached and their metabolites also ultimately accumulate in plasma. Shuttling these acylcarnitines to the plasma could potentially prevent the buildup of possibly toxic acyl-CoA species inside the cell [37]. In line with this view, we also observed increased plasma acylcarnitines concentrations in the HFD group when compared to chow, in particular long-chain acylcarnitines. COMBI treatment increased plasma medium-chain acylcarnitines compared to the HFD. The increase of these acyl carnitine species could reflect incomplete β-oxidation. However, this does not seem to be in line with our other findings that COMBI treatment attenuated obesity, fat mass gain and hepatic steatosis, which all suggest an improvement in fatty acid catabolism and β-oxidation rather than worsening. An explanation for the seemingly discordant observations is provided by Lehman et al. who showed that medium chain acylcarnitines increase after exercise and acylcarnitines may enhance lipid oxidation [38]. Although the mice in the COMBI group were not more active relative to the HFD group, they gained 17% less fat mass. Therefore, it is possible that the COMBI treatment mimics some processes that are also activated during exercise leading to a higher metabolic rate. 

The adiposity-attenuating effects of the COMBI treatment were associated with a reduction in NAFLD development, i.e., a reduction in both total steatosis and hepatocellular hypertrophy. Moreover, the liver weight was lower in mice treated with the COMBI. Collectively, the reduction in weight gain and improved liver health may be a result of improved hepatic metabolism and processing of fatty acids. Indeed, among the molecular processes underlying the observed hepatoprotective effects of the COMBI treatment were multiple pathways and upstream regulators critical for metabolic homeostasis. For example, COMBI treatment reversed HFD-induced deactivation of PPARGC1B involved in mitochondrial biogenesis and the increase of oxidative phosphorylation [39]. COMBI also reversed HFD-induced SREBF2 and SCAP modulation important players in lipid metabolism [40] and counteracted the HFD-induced inactivation of the enzyme ACOX1, which controls the first step of the peroxisomal FA β-oxidation pathway [41]. 

In conclusion, we showed that a novel treatment with a combination of LC and NR, but not the mono-treatments, significantly attenuated obesity, fat mass, hepatic steatosis and exerted beneficial effects on metabolic control pathways and upstream regulators (ACOX, SCPAP, SREBF2, PPARGC1B, INSR) in the liver. Dietary supplementation with LC and NR could constitute a new therapeutic approach to prevent obesity and its complications in the liver.

## 4. Materials and Methods 

### 4.1. Animals and Diets

All animal experiments were performed according to the rules and regulations set out by the Netherlands Law on Animal Experiments and were approved by an independent Committee on Animal Care and Experimentation (approval reference number DEC-3383, Date: 25 February 2013, DEC-Zeist, The Netherlands).

Male Ldlr−/−.Leiden mice [17,18] were bred by TNO Metabolic Health Research (Leiden, The Netherlands). Mice were housed in Makrolon^®^ cages at approximately 21 °C, with a 12-h daily light cycle and relative humidity of 50–60%. The mice were supplied with food and tap water ad libitum and mice were fed standard lab chow (Ssniff R/M diet V1530, Uden, The Netherlands) until they were 12–15 weeks old. Subsequently, 74 mice were divided into five experimental groups. The healthy reference group continued to receive a chow diet (*n* = 13) and the high-fat diet control group (HFD) received a NAFLD inducing diet (45 kcal% fat from lard, 35 kcal% from carbohydrates, primarily sucrose and 20% kcal from protein; D12451, Research Diets, New Brunswick, NJ, USA) as described previously [17] (*n* = 16). L-carnitine (0.4% *w*/*w*, Sigma-Aldrich, Zwijndrecht, The Netherlands; LC group; *n* = 15), nicotinamide riboside (0.3% *w*/*w*, NovAlix, Cedex, France; NR group; *n* = 15) and the combination of both (COMBI group, *n* = 15) were mixed through the HFD and the respective groups were treated with these diets for 21 weeks.

In order to measure ambulatory locomotor activity in the different treatment groups, mice (*n* = 8 per group) were single housed in metabolic cages (Phenomaster, TSE systems) at approximately 21 °C, with a 12-h daily light cycle for 48 h in weeks 19–21. The metabolic cages measured ambulatory locomotor activity using infrared beams along the x and y axis. Beam interruptions caused by movement of the mice were registered every 30 minutes during the 48-h timeframe and represented as counts.

### 4.2. Food Intake, Body Composition 

Food intake and body weight were monitored every three weeks of the study. Total body fat mass was assessed with EchoMRI at the start and in week 9, 15 and 21 of the study as previously described [42].

### 4.3. Plasma Measurements

Blood samples were collected after 5 h fast via tail vein bleeding for isolation of EDTA plasma for evaluation of total cholesterol, triglycerides and insulin as described [17]. Plasma HDL-cholesterol was determined after precipitation as described earlier [43] with enzymatic assays (CHOD-PAP, Roche Diagnostics, Almere, the Netherlands). Non-HDL cholesterol was calculated by subtracting HDL-cholesterol levels from total plasma cholesterol levels. Blood glucose was measured using a hand-held glucometer in tail blood during blood collection (FreeStyle Lite, Abbott, Alameda CA, USA). 

### 4.4. Sacrifice and Analysis of Fat and Liver Tissue

All animals were sacrificed under non-fasted conditions by CO2 asphyxiation after 21 weeks of dietary treatment. Blood was collected via cardiac puncture for EDTA-plasma collection. White adipose tissue (WAT) was collected from gonadal and mesenteric WAT, abdominal fat mass, and the subcutaneous fat depot. The liver was weighed and divided into different parts. The medial lobe was fixed in formalin and embedded in paraffin. Development of NAFLD was analyzed in 3-μm liver hematoxylin-eosin stained sections and analyzed by a board-certified pathologist using a well-established adapted scoring method for human NAFLD [44] as described [17]. Briefly, the level of total steatosis per mouse was expressed as a percentage of the total liver section affected by microvesicular or macrovesicular steatosis. Similarly, hypertrophy was expressed as the affected percentage of the total liver section. Steatosis and hypertrophy were scored in the liver sections at a 50× magnification. The HE stained liver sections were digitalized with a 20× magnification (Aperio AT2, Leica Biosystems, Amsterdam, The Netherlands) and used for the representative images.

### 4.5. 4-HNE Oxidative Stress-Related Marker Staining and Quantification in Liver Tissue

Oxidative stress-related marker 4-hydroxynonenal (4-HNE) was analyzed in 3-μm paraffin liver sections. After deparaffinization of the liver sections, heat-induced epitope retrieval was performed with a PH 6 citrate buffer (PT link, Dako, Denmark). Subsequently, sections were washed with 0.05% Tween-20 in phosphate-buffered saline (PBS) and blocked for 30 min at room temperature (RT) with 5% normal goat serum in PBS. Staining was performed by overnight incubation at 4 °C with Rabbit anti-4-HNE Michael adducts primary antibody (1:1000 in PBS, ref.393207, Millipore Corporation, Billerica, MA, USA PBS). The next day these sections were washed and blocked for endogenous peroxidase with 3% H2O2 during 20 min, and subsequently incubated for 45 min with a BrightVision poly-HRP-anti-rabbit secondary antibody (1:1 in PBS, Immunologic, Duiven, The Netherlands;) all at RT. Lastly, after washing the sections, Diaminobenzidine (DAB) was used for color development by 5 min incubation and nuclei were stained with hematoxylin for 30 s at RT.

The 4-HNE stained liver sections were digitalized with a 20 × magnification (Aperio AT2, Leica Biosystems, Amsterdam, The Netherlands). In these scans 4-HNE positive immunoreactivity was scored in two different liver sections in five nonoverlapping fields per liver section and expressed as amount of 4-HNE positive immunoreactivity structures per mm^2^. 

### 4.6. LC-MS/MS

Sacrifice plasma samples were used to measure L-carnitine, nicotinamide and several acylcarnitine levels. The metabolites were measured with the Metabolon analytical system (Metabolon, Inc., Durham, NC, USA). A non-targeted semiquantitative liquid chromatography–tandem mass spectrometry (LC-MS/MS) platform was applied for the identification of structurally named and unknown molecules [45,46]. All normalized relative ion counts were log transformed, and the remaining data were imputed with the minimum value on a per metabolite basis and reported in relative units (RU). 

### 4.7. Gene Expression and Pathway Analysis

Total-RNA isolation kit (Bio-connect, Huissen, The Netherlands) was used to extract RNA from snap-frozen livers. The RNA concentration was measured spectrophotometrically using a NanoDrop 1000 (Isogen Life Science, De Meern, The Netherlands). To assess the quality of the isolated RNA, the 2100 Bioanalyzer (Agilent Technologies, Amstelveen, The Netherlands) was used. Next, messenger RNA (mRNA) library was produced for next generation sequencing following the manufacturer′s protocol NEBNext Ultra Directional RNA Library Prep Kit for Illumina (New England Biolabs, Ipswich, MA, USA). The quality and yield of the PCR products were consistent with the expected size distribution (300–800 base pairs). Clustering and DNA sequencing were performed by GenomeScan BV (Leiden, The Netherlands) using a NextSeq 500 sequencer (Illumina, San Diego, CA, USA). The sequences were directly aligned with the mouse reference genome (GRCm38p4) using the Start 2.5 algorithm with default settings. Gene expression data is accessible at Gene Expression Omnibus (https://www.ncbi.nlm.nih.gov/geo/) under GSE136821 The DESeq-method [47] was used to evaluate differential expressed genes between treatment groups with a cut-off at *p* < 0.01. Effects of LC, NR and the COMBI treatment on hepatic gene expression was analyzed by gene enrichment analysis across pathways and biological processes with the Ingenuity Pathway Analysis suite (IPA; www.ingenuity.com). The upstream regulator analysis tool of IPA was used to assess the activity of upstream regulators essentially as reported in [48]. Briefly, the gene expression levels of all known target genes of an upstream regulator of interest are analyzed together. A Z-score less than -2 indicates a significantly reduced transcriptional activity, while a Z-score greater than 2 indicates a significant activation based on the direction of gene expression changes of target genes essentially as reported in translational studies [17]. In addition, the software also analyses whether the observed effects are statistically significant (*p*-value), i.e., whether a greater number of target genes are modulated than can be expected to occur by chance.

### 4.8. Statistical Analysis

All data are presented as mean ± standard error of the mean (SEM) or as indicated otherwise. This study investigated the hypothesis that L-carnitine, nicotinamide riboside and their combination attenuate the development of disease relative to the untreated HFD control group. Data distribution was tested with the Shapiro-Wilk test (*p* < 0.05) and for equal variances with Levene’s test (*p* < 0.05). For normally distributed data with equal variances, a one-way analysis of variance (ANOVA) was used, if the F-statistic was significantly different Dunnett’s post hoc test was used one-sided compared to HFD (α = 0.05). A Kruskal–Wallis test was used for data sets that were not normally distributed or did not have equal variances. If the Kruskal–Wallis test indicated a significant difference (*p* < 0.05), a Mann–Whitney U test was used to compare groups one sided to HFD. For the pathway analysis of differentially expressed genes, P values were based on Fisher′s exact test (α = 0.01).

## Figures and Tables

**Table 1:** Metabolic parameters.

	Chow	HFD	LC	NR	COMBI
Cholesterol (mM)	11.0 ± 3.3 ***	32.2 ± 14.4	28.6 ± 12.5	30.3 ± 10.6	25.4 ± 10.2
HDL-cholesterol (mM)	1.2 ± 0.6 ***	3.2 ± 1.4	2.5 ± 1.0	2.8 ± 1.3	2.5 ± 1.2
Non-HDL cholesterol (mM)	9.8 ± 3.6 ***	27.4 ± 12.3	26.1 ± 12.3	26.1 ± 8.9	22.9 ± 9.4
Triglycerides (mM)	2.6 ± 1.1 **	6.1 ± 5.4	4.9 ± 2.3	5.3 ± 3.9	3.7 ± 2.1
Glucose (mM)	7.6 ± 1.5	8.1 ± 2.1	7.5 ± 1.1	7.9 ± 1.6	7.4 ± 1.0
Insulin (ng/mL)	7.3 ± 7.1 **	24.8 ± 21.0	21.9 ± 18.7	28.0 ± 14.8	15.0 ± 7.7
ALT (U/L)	117 ± 83 ***	368 ± 249	330 ± 132	293 ± 127	267 ± 142

Blood glucose and plasma metabolic risk factors after 21 weeks of diet feeding. Values are mean ± SD, * *p* < 0.05 or ** *p* < 0.01 or *** *p* < 0.001 compared to the HFD control group.

**Table 2:** Plasma acylcarnitines.

	Acylcarnitines	HFD vs. Chow	LC vs. HFD	NR vs. HFD	COMBI vs. HFD
Short-chain	3-hydroxybutyrylcarnitine (C4-1)	0.1	0.5	−0.9	−0.5
	3-hydroxybutyrylcarnitine (C4-2)	−0.6	0.6	−0.3	0.3
	valerylcarnitine (C5)	−0.8	−0.1	−0.4	0.2
Medium-chain	hexanoylcarnitine (C6)	−0.4	−0.0	−0.5	0.4
	octanoylcarnitine (C8)	−0.2	0.3	−0.5	0.4
	decanoylcarnitine (C10)	0.3	0.7	−0.4	0.3
	laurylcarnitine (C12)	0.2	0.3	−0.5	0.3
	cis-4-decenoylcarnitine (C10:1)	0.2	0.4	−0.5	0.4
	5-dodecenoylcarnitine (C12:1)	0.3	0.3	−0.6	0.1
Long-chain	myristoylcarnitine (C14)	0.2	0.2	−0.4	0.2
	pentadecanoylcarnitine (C15)	0.8	0.2	−0.4	0.1
	palmitoylcarnitine (C16)	0.8	0.1	−0.3	−0.1
	margaroylcarnitine (C17)	1.7	0.2	−0.3	−0.0
	stearoylcarnitine (C18)	2.1	0.1	−0.2	0.0
	oleoylcarnitine (C18)	1.5	0.0	−0.4	−0.3
	arachidoylcarnitine (C20)	1.8	0.4	−0.1	0.2
	myristoleoylcarnitine (C14:1)	0.5	0.3	−0.5	0.2
	palmitoleoylcarnitine (C16:1)	0.3	0.1	−0.6	−0.4
	linoleoylcarnitine (C18:2)	0.8	0.2	−0.4	−0.1
	linolenoylcarnitine (C18:3)	0.0	0.3	−0.4	−0.0
	eicosenoylcarnitine (C20:1)	2.0	−0.1	−0.3	−0.4
	dihomo-linoleoylcarnitine (C20:2)	1.4	0.1	−0.3	−0.1
	arachidonoylcarnitine (C20:4)	0.6	0.4	−0.4	0.1
	dihomo-linolenoylcarnitine (20:3n3 or 6)	0.9	0.2	−0.3	0.0

Values present 2log transformed ratios between the groups compared and specified in the top row, positive values represent an increase and negative values a decrease. Green indicates a significant decrease, conversely, orange indicates a significant increase, with *p* < 0.05 considered as significant.

**Table 3:** Upstream regulator analysis based on hepatic gene expression of metabolism related genes.

*Upstream Regulator*	HFD vs. Chow		LC vs. HFD		NR vs. HFD		COMBI vs. HFD	
*Metabolism-related*	Activation	*p*-value	Activation	*p*-value	Activation	*p*-value	Activation	*p*-value
	z-score		z-score		z-score		z-score	
ACOX1	−7.1	2.1 × 10^−32^	n/a	n/a	n/a	n/a	2.7	7.1 × 10^−7^
ATP7B	−3.2	5.0 × 10^−6^	n/a	n/a	n/a	2.5 × 10^−2^	2.0	9.3 × 10^−4^
INSIG1	−2.1	8.1 × 10^−13^	n/a	n/a	n/a	n/a	−1.9	2.0 × 10^−5^
INSR	−2.3	6.1 × 10^−9^	n/a	n/a	n/a	n/a	1.7	1.4 × 10^−5^
NR3C1	−2.1	8.9 × 10^−18^	n/a	n/a	n/a	n/a	−1.6	3.6 × 10^−2^
PPARGC1B	−3.3	3.5 × 10^−2^	n/a	n/a	n/a	n/a	2.2	2.4 × 10^−4^
SCAP	−4.8	1.5 × 10^−9^	n/a	n/a	n/a	n/a	3.3	1.4 × 10^−11^
SREBF1	−1.1	2.0 × 10^−9^	n/a	n/a	n/a	n/a	2.7	9.7 × 10^−10^
SREBF2	−3.0	7.2 × 10^−9^	n/a	n/a	n/a	n/a	2.6	8.7 × 10^−13^
SIRT2	−3.2	9.4 × 10^−5^	n/a	n/a	n/a	n/a	2.2	1.3 × 10^−6^
TSC2	−4.7	3.9 × 10^−12^	n/a	n/a	n/a	n/a	2.0	8.9 × 10^−3^
CDKN2A	0.0	2.7 × 10^−9^	n/a	n/a	n/a	n/a	−2.4	4.0 × 10^−2^
CNR1	2.7	7.4 × 10^−3^	n/a	n/a	n/a	n/a	n/a	n/a
CYP51A1	3.0	1.8 × 10^−5^	n/a	n/a	n/a	n/a	n/a	3.7 × 10^−4^
CYP2E1	2.1	6.2 × 10^−6^	n/a	n/a	n/a	n/a	n/a	n/a
EP300	4.5	8.7 × 10^−14^	n/a	n/a	n/a	n/a	−2.2	2.7 × 10^−3^
FOXO1	4.5	4.1 × 10^−9^	n/a	n/a	n/a	n/a	−2.1	1.5 × 10^−2^
FOXO3	0.6	9.2 × 10^−7^	n/a	n/a	n/a	n/a	−2.2	4.3 × 10^−2^
MAT1A	2.4	9.0 × 10^−2^	n/a	n/a	n/a	n/a	n/a	n/a
NR3C2	2.1	8.9 × 10^−7^	n/a	n/a	n/a	n/a	−2.0	6.4 × 10^−3^
TP53	2.2	1.58 × 10^−60^	n/a	n/a	n/a	n/a	−2.0	2.0 × 10^−3^

Changes in upstream regulator are predicted from changes in transcription factors or key regulators with a Z-score. Z < −2 indicates a relevant inhibition (shown in orange) and Z > 2 indicates a relevant activation (shown in green). Significant changes with Z > 1.5 are shown in light orange. The *p*-value indicates significant enrichment of the genes downstream of a regulator (*p* < 0.01 was considered statistically significant). n/a indicates an insufficient number of differentially expressed genes to link gene effects to an upstream regulator.

## Supplementary Materials

Supplementary materials can be found at https://www.mdpi.com/1422-0067/20/18/4359/s1.

## Abbreviations

NAFLD	Non-alcoholic fatty liver disease
LC	L-Carnitine
NAD+	Nicotinamide adenine dinucleotide
NR	Nicotinamide riboside
FA	Fatty acid
4-HNE	4-Hydroxynonenal
COMBI	Combination of LC and NR treatment in this study
Ldlr-/-	Low-density lipoprotein receptor knockout
HFD	High-fat diet
BW	Body weight
FI	Food intake
WAT	White adipose tissue
HDL	High-density lipoprotein
ALT	Alanine aminotransferase
DEG	Differentially expressed genes
RU	Relative units

## References

[1] Ng M., Fleming T., Robinson M., Thomson B., Graetz N., Margono C., Mullany E.C., Biryukov S., Abbafati C., Abera S.F. (2014). Global, regional, and national prevalence of overweight and obesity in children and adults during 1980-2013: A systematic analysis for the Global Burden of Disease Study 2013. Lancet.

[2] Younossi Z.M., Koenig A.B., Abdelatif D., Fazel Y., Henry L., Wymer M. (2016). Global epidemiology of nonalcoholic fatty liver disease—Meta-analytic assessment of prevalence, incidence, and outcomes. Hepatology.

[3] Fildes A., Charlton J., Rudisill C., Littlejohns P., Prevost A.T., Gulliford M.C. (2015). Probability of an obese person attaining normal body weight: Cohort study using electronic health records. Am. J. Public Health.

[4] Lee Y., Kwon E.Y., Choi M.S. (2018). Dietary isoliquiritigenin at a low dose ameliorates insulin resistance and NAFLD in diet-induced obesity in C57BL/6J mice. Int. J. Mol. Sci..

[5] Schrader M., Costello J., Godinho L.F., Islinger M. (2015). Peroxisome-mitochondria interplay and disease. J. Inherit. Metab. Dis..

[6] Wijburg F.A., Wüst R.C.I., Visser G., Wanders R.J.A., Knottnerus S.J.G., IJlst L., Houtkooper R.H., Ferdinandusse S., Bleeker J.C. (2018). Disorders of mitochondrial long-chain fatty acid oxidation and the carnitine shuttle. Rev. Endocr. Metab. Disord..

[7] Antonenkov V.D., Hiltunen J.K. (2012). Transfer of metabolites across the peroxisomal membrane. Biochim. Biophys Acta. – Mol. Basis. Dis..

[8] Noland R.C., Koves T.R., Seiler S.E., Lum H., Lust R.M., Ilkayeva O., Stevens R.D., Hegardt F.G., Muoio D.M. (2009). Carnitine insufficiency caused by aging and overnutrition compromises mitochondrial performance and metabolic control. J. Biol. Chem..

[9] Cantó C., Houtkooper R.H., Pirinen E., Youn D.Y., Oosterveer M.H., Cen Y., Fernandez-Marcos P.J., Yamamoto H., Andreux P.A., Cettour-Rose P. (2012). The NAD+ precursor nicotinamide riboside enhances oxidative metabolism and protects against high-fat diet-induced obesity. Cell Metab..

[10] Garten A., Schuster S., Penke M., Gorski T., De Giorgis T., Kiess W. (2015). Physiological and pathophysiological roles of NAMPT and NAD metabolism. Nat. Rev. Endocrinol..

[11] Yoshino J., Baur J.A., Imai S.I. (2018). NAD + Intermediates: The Biology and Therapeutic Potential of NMN and NR. Cell Metab..

[12] Bournat J.C., Brown C.W. (2016). Mitochondrial Dysfunction in Obesity. Curr. Opin. Endocrino.l Diabetes Obes..

[13] Zhong H., Yin H. (2015). Role of lipid peroxidation derived 4-hydroxynonenal (4-HNE) in cancer: Focusing on mitochondria. Redox. Biol..

[14] Loguercio C., De Girolamo V., De Sio I., Tuccillo C., Ascione A., Baldi F., Budillon G., Cimino L., Di Carlo A., Pia Di Marino M. (2001). Non-alcoholic fatty liver disease in an area of southern italy: Main clinical, histological, and pathophysiological aspects. J. Hepatol..

[15] Seki S., Kitada T., Yamada T., Sakaguchi H., Nakatani K., Wakasa K. (2002). In situ detection of lipid peroxidation and oxidative DNA damage in non-alcoholic fatty liver diseases. J. Hepatol..

[16] Ore A., Akinloye O.A. (2019). Oxidative Stress and Antioxidant Biomarkers in Clinical and Experimental Models of Non-Alcoholic Fatty Liver Disease. Medicina.

[17] Morrison M.C., Verschuren L., Salic K., Verheij J., Menke A., Wielinga P.Y., Iruarrizaga-Lejarreta M., Gole L., Yu W., Turner S. (2018). Obeticholic Acid Modulates Serum Metabolites and Gene Signatures Characteristic of Human NASH and Attenuates Inflammation and Fibrosis Progression in Ldlr-/-.Leiden Mice. Hepatol. Commun..

[18] Morrison M.C., Kleemann R., van Koppen A., Hanemaaijer R., Verschuren L. (2018). Key inflammatory processes in human NASH are reflected in Ldlr-/-.Leiden mice: A translational gene profiling study. Front. Physiol..

[19] Van Koppen A., Verschuren L., van den Hoek A.M., Verheij J., Morrison M.C., Li K., Nagabukuro H., Costessi A., Caspers M.P.M., van den Broek T.J. (2018). Uncovering a Predictive Molecular Signature for the Onset of NASH-Related Fibrosis in a Translational NASH Mouse Model. Cell Mol. Gastroenterol. Hepatol..

[20] Ishikawa H., Takaki A., Tsuzaki R., Yasunaka T., Koike K., Shimomura Y., Seki H., Matsushita H., Miyake Y., Ikeda F. (2014). L-carnitine prevents progression of non-alcoholic steatohepatitis in a mouse model with upregulation of mitochondrial pathway. PLoS ONE.

[21] Melton S.A., Keenan M.J., Stanciu C.E., Hegsted M., Zablah-Pimentel E.M., O’Neil C.E., Gaynor P., Schaffhauser A., Owen K., Prisby R.D. (2005). L-carnitine supplementation does not promote weight loss in ovariectomized rats despite endurance exercise. Int. J. Vitam. Nutr. Res..

[22] Fujisawa K., Takami T., Matsuzaki A., Matsumoto T., Yamamoto N., Terai S., Sakaida I. (2017). Evaluation of the effects of L-carnitine on medaka (Oryzias latipes) fatty liver. Sci. Rep..

[23] Del Vecchio F., Coswig V., Galliano L. (2016). Comment on ‘The effect of (L-)carnitine on weight loss in adults: A systematic review and meta-analysis of randomized controlled trials. Obes. Rev..

[24] Shi W., Hegeman M.A., van Dartel D.A.M., Tang J., Suarez M., Swarts H., van der Hee B., Arola L., Keijer J. (2017). Effects of a wide range of dietary nicotinamide riboside (NR) concentrations on metabolic flexibility and white adipose tissue (WAT) of mice fed a mildly obesogenic diet. Mol. Nutr. Food Res..

[25] Martens C.R., Denman B.A., Mazzo M.R., Armstrong M.L., Reisdorph N., McQueen M.B., Chonchol M., Seals D.R. (2018). Chronic nicotinamide riboside supplementation is well-Tolerated and elevates NAD+ in healthy middle-Aged and older adults. Nat. Commun..

[26] Dollerup O.L., Christensen B., Svart M., Schmidt M.S., Sulek K., Ringgaard S., Stødkilde-Jørgensen H., Møller N., Brenner C., Treebak J.T. (2018). A randomized placebo-controlled clinical trial of nicotinamide riboside in obese men: Safety, insulin-sensitivity, and lipid-mobilizing effects. Am. J. Clin. Nutr..

[27] Villani R.G., Gannon J., Self M., Rich P.A. (2016). L-Carnitine Supplementation Combined with Aerobic Training Does Not Promote Weight Loss in Moderately Obese Women. Int. J. Sport. Nutr. Exerc. Metab..

[28] Rafraf M., Karimi M., Jafari A. (2015). Effect of L-carnitine supplementation in comparison with moderate aerobic training on serum inflammatory parameters in healthy obese women. J. Sports Med. Phys. Fitness..

[29] Gariani K., Menzies K.J., Ryu D., Wegner C.J., Wang X., Ropelle E.R., Moullan N., Zhang H., Perino A., Lemos V. (2016). Eliciting the mitochondrial unfolded protein response by nicotinamide adenine dinucleotide repletion reverses fatty liver disease in mice. Hepatology.

[30] Watanabe H., Inaba Y., Kimura K., Matsumoto M., Kaneko S., Kasuga M., Inoue H. (2018). Sirt2 facilitates hepatic glucose uptake by deacetylating glucokinase regulatory protein. Nat. Commun..

[31] Malaguarnera M., Gargante M.P., Russo C., Antic T., Vacante M., Malaguarnera M., Avitabile T., Li Volti G., Galvano F. (2010). L-carnitine supplementation to diet: A new tool in treatment of nonalcoholic steatohepatitisa randomized and controlled clinical trial. Am. J. Gastroenterol..

[32] Ruggenenti P., Cattaneo D., Loriga G., Ledda F., Motterlini N., Gherardi G., Orisio S., Remuzzi G. (2009). Ameliorating hypertension and insulin resistance in subjects at increased cardiovascular risk: Effects of acetyl-l-carnitine therapy. Hypertension.

[33] Cheema U.B., Most E., Eder K., Ringseis R. (2019). Effect of lifelong carnitine supplementation on plasma and tissue carnitine status, hepatic lipid metabolism and stress signalling pathways and skeletal muscle transcriptome in mice at advanced age. Br. J. Nutr..

[34] Malaguarnera M., Gargante M.P., Russo C., Antic T., Vacante M., Malaguarnera M., Avitabile T., Li Volti G., Galvano F. (2010). Increased levels of plasma acylcarnitines in obesity and type 2 diabetes and identification of a marker of glucolipotoxicity. Obesity.

[35] Koves T.R., Li P., An J., Akimoto T., Slentz D., Ilkayeva O., Dohm G.L., Yan Z., Newgard C.B., Muoio D.M. (2005). Peroxisome proliferator-activated receptor-γ co-activator 1α-mediated metabolic remodeling of skeletal myocytes mimics exercise training and reverses lipid-induced mitochondrial inefficiency. J. Biol. Chem..

[36] Koves T.R., Ussher J.R., Noland R.C., Slentz D., Mosedale M., Ilkayeva O., Bain J., Stevens R., Dyck J.R.B., Newgard C.B. (2008). Mitochondrial Overload and Incomplete Fatty Acid Oxidation Contribute to Skeletal Muscle Insulin Resistance. Cell Metab..

[37] Li L.O., Klett E.L., Coleman R.A. (2010). Acyl-CoA synthesis, lipid metabolism and lipotoxicity. Biochim. Biophys. Acta. – Mol. Cell Biol. Lipids.

[38] Lehmann R., Zhao X., Weigert C., Simon P., Fehrenbach E., Fritsche J., Machann J., Schick F., Wang J., Hoene M. (2010). Medium chain acylcarnitines dominate the metabolite pattern in humans under moderate intensity exercise and support lipid oxidation. PLoS ONE.

[39] St-Pierre J., Lin J., Krauss S., Tarr P.T., Yang R., Newgard C.B., Spiegelman B.M. (2003). Bioenergetic analysis of peroxisome proliferator-activated receptor γ coactivators 1α and 1β (PGC-1α and PGC-1β) in muscle cells. J. Biol. Chem..

[40] Cheng X., Li J., Guo D. (2018). SCAP/SREBPs are Central Players in Lipid Metabolism and Novel Metabolic Targets in Cancer Therapy. Curr. Top Med. Chem..

[41] Hu T., Foxworthy P., Siesky A., Ficorilli J.V., Gao H., Li S., Christe M., Ryan T., Cao G., Eacho P. (2005). Hepatic peroxisomal fatty acid β-oxidation is regulated by liver X receptor α. Endocrinology.

[42] Choemaker M.H., Kleemann R., Morrison M.C., Verheij J., Salic K., Van Tol E.A.F., Kooistra T., Wielinga P.Y. (2017). A casein hydrolysate based formulation attenuates obesity and associated nonalcoholic fatty liver disease and atherosclerosis in LDLr-/-.Leiden mice. PLoS ONE.

[43] Kühnast S., Van Der Tuin S.J.L., Van Der Hoorn J.W.A., Van Klinken J.B., Simic B., Pieterman E., Havekes L.M., Landmesser U., Lüscher T.F., Van Dijk K.W. (2015). Anacetrapib reduces progression of atherosclerosis, mainly by reducing non-HDL-cholesterol, improves lesion stability and adds to the beneficial effects of atorvastatin. Eur. Heart J..

[44] Liang W., Menke A.L., Driessen A., Koek G.H., Lindeman J.H., Stoop R., Havekes L.M., Kleemann R., Van Den Hoek A.M. (2014). Establishment of a general NAFLD scoring system for rodent models and comparison to human liver pathology. PLoS ONE.

[45] Evans A.M., DeHaven C.D., Barrett T., Mitchell M., Milgram E. (2009). Integrated, nontargeted ultrahigh performance liquid chromatography/ electrospray ionization tandem mass spectrometry platform for the identification and relative quantification of the small-molecule complement of biological systems. Anal. Chem..

[46] Evans A., Bridgewater B., Liu Q., Mitchell M., Robinson R., Dai H., Stewart S., DeHaven C., Miller L. (2015). High Resolution Mass Spectrometry Improves Data Quantity and Quality as Compared to Unit Mass Resolution Mass Spectrometry in High-Throughput Profiling Metabolomics. J. Postgenomics Drug Biomark Dev..

[47] Anders S., Huber W. (2010). Differential expression analysis for sequence count data. Genome Biol..

[48] Liang W., Tonini G., Mulder P., Kelder T., van Erk M., van den Hoek A.M., Mariman R., Wielinga P.Y., Baccini M., Kooistra T. (2013). Coordinated and Interactive Expression of Genes of Lipid Metabolism and Inflammation in Adipose Tissue and Liver during Metabolic Overload. PLoS ONE.

