# Supplementary material for: Combined Treatment with L-Carnitine and Nicotinamide Riboside Improves Hepatic Metabolism and Attenuates Obesity and Liver Steatosis
Source: Int J Mol Sci. 2019 Sep 5;20(18):4359. doi: 10.3390/ijms20184359 (PMC6770226; doi:10.3390/ijms20184359)
Supplement: Supplementary file 1 [file ijms-20-04359-s001.zip › ijms-569120.-supplementary/Supplemental table 1 LC and NR paper_24072019.pdf]

Supplemental table 1

Chemokines	HFD vs Chow		LC vs HFD		NR vs HFD		COMBI vs HFD	
	z-score	p-value	z-score	p-value	z-score	p-value	z-score	p-value
CCL2	1,4	3,4E-04	na	na	na	na	na	na
CCL5	2,6	8,0E-10	na	na	na	na	na	na
Ccl6	2,4	7,1E-04	na	na	na	na	na	na
CXCL2	2,5	1,9E-04	na	na	na	na	na	na
Cxcl3	3,1	2,3E-05	na	na	na	na	na	na
CXCL3	3,1	2,3E-05	na	na	na	na	na	na
CXCL12	3,7	1,8E-09	na	na	na	na	na	na

Cytokines	HFD vs Chow		LC vs HFD		NR vs HFD		COMBI vs HFD	
	z-score	p-value	z-score	p-value	z-score	p-value	z-score	p-value
IL1A	5,5	7,0E-13	na	na	na	na	0,1	3,7E-02
IL1B	6,9	1,8E-31	na	na	-1,7	1,9E-02	-0,2	1,6E-03
IL1RN	-4,2	3,8E-05	na	na	na	na	na	na
IL2	6,0	6,0E-21	na	na	na	na	na	na
Il3	-0,7	2,3E-03	na	na	na	na	-1,0	4,3E-03
IL4	3,6	4,4E-26	na	na	na	na	-2,0	5,8E-04
IL5	5,0	3,4E-18	na	na	na	na	na	na
IL6	3,5	4,4E-35	na	na	-1,8	7,4E-02	-1,0	5,6E-03
IL7	2,2	5,2E-05	na	na	na	na	na	na
IL9	2,4	4,8E-01	na	na	na	na	na	na
IL10	1,0	2,6E-14	na	na	na	na	-2,8	6,4E-04
IL10RA	-3,9	1,2E-32	0,0	4,4E-02	na	na	na	na
IL12B	2,1	3,4E-03	na	na	na	na	na	na
IL13	4,0	5,3E-26	na	na	na	na	-1,6	3,7E-04
IL15	4,2	2,7E-12	na	na	na	na	na	na
IL17A	3,3	8,1E-13	na	na	-0,9	3,9E-02	0,2	2,7E-02
IL18	3,8	9,7E-04	na	na	na	na	na	na
IL21	3,4	2,2E-07	na	na	na	na	na	na
IL27	4,1	7,1E-04	na	na	na	na	na	na
IFNA2	4,8	1,3E-03		1,5E-02	na	na	na	na
IFNA4	na	na	na	na	na	na	na	na
IFNB1	4,5	1,8E-14	na	na	na	na		3,1E-02
IFNE	2,4	1,8E-03	na	na	na	na	na	na
IFNG	7,2	4,9E-50	na	na	na	na	0,2	4,9E-02
IFNL1	3,3	3,0E-03	na	na	na	na	na	na
IFNL3	2,4	2,4E-02	na	na	na	na	na	na
IFNL3	2,4	2,4E-02	na	na	na	na	na	na
IFNL3	2,4	2,4E-02	na	na	na	na	na	na
IFNL3	2,4	2,4E-02	na	na	na	na	na	na
CSF1	4,5	5,1E-18	na	na	na	na	-1,2	4,3E-02
CSF2	7,2	1,2E-36	na	na	na	na	na	na
CSF3	2,3	7,9E-19	na	na	na	na	na	na
MIF	3,3	9,0E-04	na	na	na	na	na	na
OSM	2,4	5,1E-17	na	na	na	na	0,2	1,7E-02
TNF	7,2	2,3E-46	na	na	na	na	-0,3	4,2E-04
TNFSF11	4,9	5,4E-23	na	na	-0,4	2,1E-02	-2,4	2,3E-02

TLR-signalling	HFD vs Chow		LC vs HFD		NR vs HFD		COMBI vs HFD	
	z-score	p-value	z-score	p-value	z-score	p-value	z-score	p-value
TLR2	3,0	7,3E-04	na	na	na	na	na	na
TLR3	4,7	2,5E-07	na	na	na	na	na	na
TLR4	5,0	8,6E-15	na	na	na	na	na	na
TLR5	3,1	7,2E-03	na	na	na	na	na	na
TLR7	5,5	7,3E-05	na	na	na	na	na	na
TLR9	4,5	1,9E-09	na	na	na	na	na	na
MYD88	5,5	5,8E-14	na	na	na	na	na	na

Inflammation	HFD vs Chow		LC vs HFD		NR vs HFD		COMBI vs HFD	
	z-score	p-value	z-score	p-value	z-score	p-value	z-score	p-value
JUN	4,4	4,4E-21	na	na	na	na	0,1	8,0E-03
JUNB	3,9	1,4E-06	na	na	na	na		1,3E-02
JUND	0,7	5,4E-05	na	na	na	na	na	na
ITK	3,0	1,5E-05	na	na	na	na	na	na
MAPK8	2,3	8,5E-05	na	na	na	na	na	na
Nfat (family)	4,2	3,7E-05	na	na	na	na	na	na
NFAT5	na	1,1E-02	na	na	na	na	na	na
NFATC1	2,0	6,6E-04	na	na	na	na	na	na
NFATC2IP	na	na	na	na	na	na	na	na
NFATC3	na	na	na	na	na	na	na	na
NFATC4	na	na	na	na	na	na	na	na
NFATC2	2,4	6,7E-05	na	na	na	na	na	na
NFkB (family)	2,0	2,5E-03	na	na	na	na	na	1,0E-02
NFKB1	3,9	2,4E-09	na	na	na	na	na	na
NFKB2	0,1	2,2E-02	na	na	na	na	na	na
RELA	3,9	7,3E-08	na	na	na	na	na	na
NFKBIA	3,8	6,9E-18	na	na	na	na	na	na
NFKB1	3,9	2,4E-09	na	na	na	na	na	na
STAT1	6,1	4,8E-16	na	na	na	na	na	na
STAT3	3,0	1,6E-25	na	na	na	na	-0,9	3,3E-02
STAT4	5,1	5,0E-08	na	na	na	na	na	na
STAT5A	0,9	4,3E-08	na	na	na	na	-2,2	9,5E-02
STAT5B	0,2	1,2E-04	na	na	na	na	-2,0	3,9E-02
SIRT2	-3,2	9,4E-05	na	na	na	na	2,2	1,3E-06
HNF1A	-2,9	2,3E-12	na	1,2E-02	1,7	1,1E-04	0,8	4,0E-05
Irgm1	-4,3	4,4E-08	na	na	na	na	na	na

Oxidative stress response	HFD vs Chow		LC vs HFD		NR vs HFD		COMBI vs HFD	
	z-score	p-value	z-score	p-value	z-score	p-value	z-score	p-value
Nos	na	na	na	na	na	na	na	na
NOS2	2,9	6,45E-11	na	na	na	na	-0,9	1,82E-02
NOS3	-0,3	1,59E-06	na	na	na	na	na	na
CYP2E1	2,1	6,19E-06	na	na	na	na	na	na
NADPH oxidase	2,4	1,82E-02	na	na	na	na	na	na
NCF2	na	na	na	na	na	na	na	na
NOX1	na	na	na	na	na	na	na	na
NOX3	na	na	na	na	na	na	na	na
NOX4	1,3	1,29E-02	na	na	na	na	na	2,49E-02
GPX1	-2,4	2,63E-05	na	na	na	na	na	na
COL18A1	-3,4	1,28E-10	na	na	na	na	na	na
SOD	-2,6	7,03E-04	na	na	na	na	na	na
SOD1	-1,2	8,64E-08	na	na	na	na	na	2,50E-02
SOD2	1,0	1,12E-05	na	na	na	na	na	2,37E-02
SOD3	na	na	na	na	na	na	na	na
NFE2L2 (NRF2)	na	na	na	na	na	na	na	na
POR	1,5	3,89E-24	na	na	na	5,68E-03	-2,0	2,00E-09
DUSP1	-0,2	2,12E-13	na	na	na	na	na	na
CAT	-2,8	7,81E-03	na	na	na	na	na	na
GSTZ1	na	2,35E-02	na	na	na	na	na	na
GSTA1	na	na	na	na	na	na	na	2,23E-02
