# Supplementary figures and images for: Combined Treatment with L-Carnitine and Nicotinamide Riboside Improves Hepatic Metabolism and Attenuates Obesity and Liver Steatosis
Source: Int J Mol Sci. 2019 Sep 5;20(18):4359. doi: 10.3390/ijms20184359 (PMC6770226; doi:10.3390/ijms20184359)

Supplemental figure 1

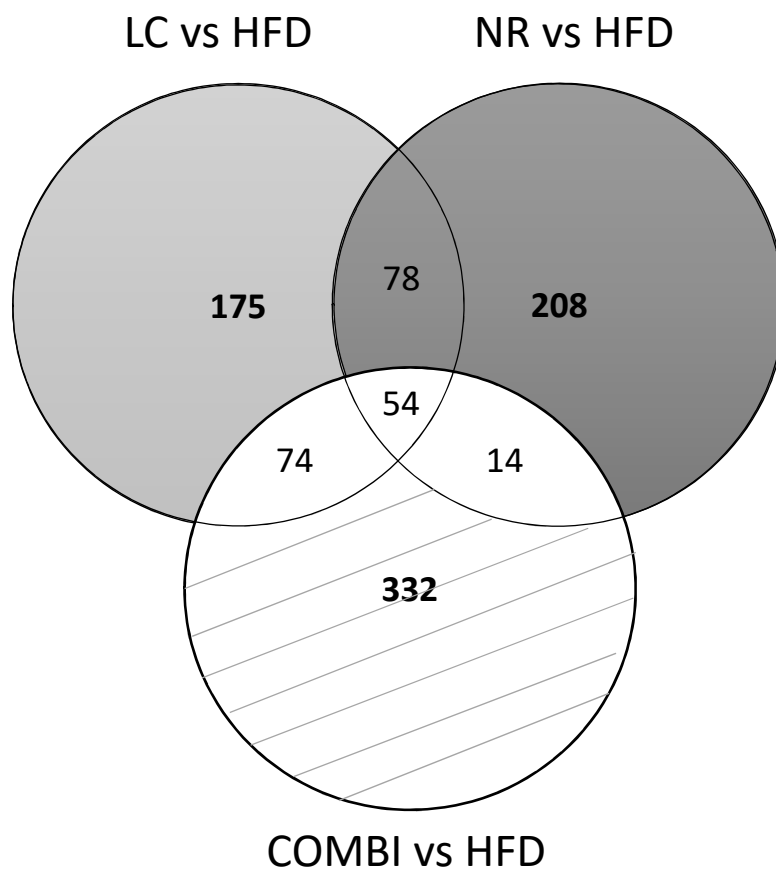

Supplement: Supplementary file 1 [file ijms-20-04359-s001.zip › ijms-569120.-supplementary/Supplemental Figure 1 LC and NR paper_24072019.pdf]
